# Supplementary material for: Sex‐Specific doublesex Regulation Targeting the Color‐Patterning Gene h Underlies the Evolution of Wing Sexual Dimorphism in the Harlequin Ladybug Harmonia axyridis
Source: Evol Dev. 2026 Jan 6;28(1):e70028. doi: 10.1111/ede.70028 (PMC12771471; doi:10.1111/ede.70028)
Supplement: Supplementary file 1 — Fig. S1. Multiple alignment of the protein sequences of DMRT‐type transcription factors in Insecta and Vertebrata. Multiple sequence alignments were generated for the amino acid sequences of Dsx and the other Dmrt family transcription factors. Poorly aligned regions were removed using the TrimAl program. Black boxes indicate conserved residues shared by more than 80% of taxa. The region marked with a red bar corresponds to the DM domain, which is highly conserved among DMRT‐type transcription factors. [file EDE-28-e70028-s004.pdf]

```
                     1 ........10........20........30........40........50........60
Haxy_Dsx             1 --MS--------------DSQEFEIKTDT-------------------------------
Dmel_Dsx             1 M-VS--EE-NWN-SDTMSDSDMIDSKNDVCGG----------------------------
Lcup_Dsx             1 M-VS--EDTNWNSSDTMSDTDMHDSKNDICGG----------------------------
Tcas_Dsx             1 --MS-------------SDSQDFDSKMDV-------------------------------
Bmor_Dsx             1 M-VS--MG-SW----KRRVPDDCEERSEP-------------------------------
Ekue_Dsx             1 M-VS--VG-AW----RRRAPDDCEERSEP-------------------------------
Tdom_Dsx             1 M-MADNQDTNLSSPETVVGVDNSPPEGLENGGL---------------------------
Dmel_Dmrt99B         1 --MS--LP-------SGVDMQNLMSQHPVLGALPP----------AFFLR----------
Lcup_Dmrt99B         1 --MS--LP-------SGVDMQNLMSQHPVLGALPP----------AFFLR----------
Tcas_Dmrt99B         1 --MS--LP------SSGVDMSSLMSQHPVLGAIPP----------AFFLR----------
Bmor_Dmrt99B         1 MEMS--LP-------NGVDVSQLV-QHPVLAALPP-----------FFLR----------
Bmut_Dmrt1           1 --MP--ND-------DAYSKPSAPSEAPQTPGAPPQGKAGG-----------------GG
Drer_Dmrt1           1 --MS--EE-------E--------------------------------------------
Mmus_Dmrt1           1 --MP--ND-------DTFGKPSTPTEVPHAPGAPPQGKAGGYSKAAGAMAGAAGGSGAGG
Xlae_Dmrt1           1 --MQ-NNE-------EPYSKTRNSGQHPS-------------------------------

                                                DM domain (Not trimmed)
                    61 ........70........80........90........100.......110.......120
Haxy_Dsx            14 -----------NASSTSTNPRTPPNCARCRNHRMKIPLKGHKRYCKYRNCACEKCRLTSE
Dmel_Dsx            28 ---------ASSSSGSSISPRTPPNCARCRNHGLKITLKGHKRYCKFRYCTCEKCRLTAD
Lcup_Dsx            30 ---------ASSSSGSSGTPRTKPNCARCHNHGFKIKLKGHKRYCKYRNCNCEKCRLTAD
Tcas_Dsx            15 -----------NASSTSASPRTPPNCARCRNHRLKIALKGHKRYCKYRTCKCEKCRLTTE
Bmor_Dsx            22 ------------GASSSGVPRAPPNCARCRNHRLKIELKGHKRYCKYQHCTCEKCRLTAD
Ekue_Dsx            22 ------------GATSSGVPRAPPNCARCRNHRLKIELKGHKRYCKYRNCMCEKCRLTAD
Tdom_Dsx            33 ------------GTSSGQNARTPPKCARCRNHRLKIPLKGHKRYCKYRFCNCDKCLLTAE
Dmel_Dmrt99B        30 -------------AAS-ERYQRTPKCARCRNHGVVSALKGHKRYCRWRDCVCAKCTLIAE
Lcup_Dmrt99B        30 -------------AAS-ERYQRTPKCARCRNHGVVSALKGHKRYCRWRDCLCAKCTLIAE
Tcas_Dmrt99B        31 --------------AS-ERYQRTPKCARCRNHGVVSALKGHKRYCRWRDCNCAKCTLIAE
Bmor_Dmrt99B        30 --------------AS-ERYQRTPKCARCRNHGVVSALKGHKRYCRWRDCVCAKCTLIAE
Bmut_Dmrt1          33 GGGGSDSGASGTGAVSGKKSPRLPKCARCRNHGYASPLKGHKRFCMWRDCQCKKCNLIAE
Drer_Dmrt1           6 ---------QTNGSLSIRKPSRMPKCSRCRNHGFVSPLKGHKRFCNWRDCQCQKCRLIAE
Mmus_Dmrt1          50 SGGASGSGPSGLGSGS-KKSPRLPKCARCRNHGYASPLKGHKRFCMWRDCQCKKCSLIAE
Xlae_Dmrt1          20 ------------GVHT-KKSPRLPKCARCRNHGYASPLKGHKRYCMWRDCQCKKCSLIAE

                   121 .......130.......140.......150.......160.......170.......180
Haxy_Dsx            63 RQRVMAMQTALRRAQAQDEAMAKSG-----------------------------------
Dmel_Dsx            79 RQRVMALQTALRRAQAQDEQRALHMHEVPPANPAATTLLSHHHHVAAPAHVHAHHVHAHH
Lcup_Dsx            81 RQRVMALQTALRRAQQQDEQRILQMHEVPPVVHPPPALLKAHYH-------HHHHLQHHL
Tcas_Dsx            64 RQRVMAMQTALRRAQAQDEAMLRSG-----------------------------------
Bmor_Dsx            70 RQRVMAKQTAIRRAQAQDEARARAL-----------------------------------
Ekue_Dsx            70 RQRVMALQTALRRAQAQDEARARS------------------------------------
Tdom_Dsx            81 RQRVMALQTALRRAQAQDEARAAGQ--IP-------------------------------
Dmel_Dmrt99B        76 RQRVMAAQVALRRQQAQEENEAREL-----------------------------------
Lcup_Dmrt99B        76 RQRVMAAQVALRRQQAQEENEAREL-----------------------------------
Tcas_Dmrt99B        76 RQRVMAAQVALRRQQAQEENEAREL-----------------------------------
Bmor_Dmrt99B        75 RQRVMAAQVALRRQQAQEENEAREL-----------------------------------
Bmut_Dmrt1          93 RQRVMAAQVALRRQQAQEE----EL-----------------------------------
Drer_Dmrt1          57 RQRVMAAQVALRRQQAQEE----EM-----------------------------------
Mmus_Dmrt1         109 RQRVMAAQVALRRQQAQEE----EL-----------------------------------
Xlae_Dmrt1          67 RQRVMAAQVALRRQQAQEE----EL-----------------------------------

                   181 .......190.......200.......210.......220.......230.......240
Haxy_Dsx            88 ------------------------AID---------------------------------
Dmel_Dsx           139 AHGGHHSHHGHVLHHQQAAAAAAAAPSAPASHLGGSSTAASSIHGHAHAHHVHMAAAAAA
Lcup_Dsx           134 AEQLHHHHHPHLVDATTSAAVVGAAVP---------------------------------
Tcas_Dsx            89 -----------------------SAVD---------------------------------
Bmor_Dsx            95 ---------------ELGIQPPGLELD---------------------------------
Ekue_Dsx            94 -----------------GVQANGVELD---------------------------------
Tdom_Dsx           108 -----------------GAQS---------------------------------------
Dmel_Dmrt99B       101 -----------------GLLYTSVP-----------------------------------
Lcup_Dmrt99B       101 -----------------GLLYTSVPPR---------------------------------
Tcas_Dmrt99B       101 -----------------GILF---------------------------------------
Bmor_Dmrt99B       100 -----------------NLLYAGQPSS---------------------------------
Bmut_Dmrt1         114 -----------------GISH---------------------------------------
Drer_Dmrt1          78 -----------------GICS---------------------------------------
Mmus_Dmrt1         130 -----------------GISH---------------------------------------
Xlae_Dmrt1          88 -----------------GISH---------------------------------------
```

```
                241 .......250.......260.......270.......280.......290.......300
Haxy_Dsx         91 ----------------------------PHILQ------NTPSPI---------------
Dmel_Dsx        199 SVAQHQHQSHPHSHHHHHQNHHQHPHQQPATQTAL----RSPPHSDHGGSVGPATSSSGG
Lcup_Dsx        161 ----------PHHHHHHHVTH----AAAAAAISTI----RSPPHSDH--SVNGGSSAGGG
Tcas_Dsx         93 ----------------------------PAIMQVP---LKSPSPIH--------------
Bmor_Dsx        107 -------------------------RPVPPVVKAP----RSPMIPP--------------
Ekue_Dsx        104 -------------------------RPDPPAVKTQ----RSPVPP---------------
Tdom_Dsx        112 ----------------------------PVDMESSAGASSIPGPS---------------
Dmel_Dmrt99B    109 -------------------------------GQQN---GSDSATPTP-------------
Lcup_Dmrt99B    111 ---------------------------VPSQNTNE---NTTTATTSQ-------------
Tcas_Dmrt99B    105 ----------------------------PTPAGVV---ADTPGVTAP-------------
Bmor_Dmrt99B    110 ---------------------------AP-------------------------------
Bmut_Dmrt1      118 ----------------------------PIPL---------PSTAEL-------------
Drer_Dmrt1       82 ----------------------------PINL----------SGSDT-------------
Mmus_Dmrt1      134 ----------------------------PIPL---------PSAAEL-------------
Xlae_Dmrt1       92 ----------------------------PIHL---------PIAAEL-------------

                301 .......310.......320.......330.......340.......350.......360
Haxy_Dsx        102 --------------------------------------LLKRKL-------DCDSSSSSQ
Dmel_Dsx        255 GAPSSSNAAAATSSNGSSGGGGGGGGGSSGGG-------------AGGGRSSGTSVITSA
Lcup_Dsx        201 G-----------------GNNGGGGGGSAGGGVVSSVGAIERNAAALNGMASSSSIASSS
Tcas_Dsx        108 --------------------------------------AIERSL-------DCDSSASSQ
Bmor_Dsx        124 --------------------------------------SAPRSL----GSASCDSVPGSP
Ekue_Dsx        120 ----------------------------------------PRSL----GSASCDSVPGSP
Tdom_Dsx        129 --------------------------------------SLSRE--TVVSAGGCDSSSTSP
Dmel_Dmrt99B    122 --------------------------------------HSPNHSGSGGSGSGGSGQNGVF
Lcup_Dmrt99B    128 --------------------------------------LSPQQTSA----------NGVF
Tcas_Dmrt99B    121 --------------------------------------AIPQ---NSDVGISQLMQRNTF
Bmor_Dmrt99B    112 ------------------------------------------------------------
Bmut_Dmrt1      128 --------------------------------------MVKR---ENSSGNPCLMIESSS
Drer_Dmrt1       91 --------------------------------------LVKN---EAVGEN-VFTLSSGP
Mmus_Dmrt1      144 --------------------------------------LVKR---ENNASNPCLMAENSS
Xlae_Dmrt1      102 --------------------------------------LIKK---EHGGSSSCLMLENSS

                361 .......370.......380.......390.......400.......410.......420
Haxy_Dsx        117 CSPP--------------------------------------------------------
Dmel_Dsx        302 ------------------DHHM--------------------------------------
Lcup_Dsx        244 TAGPPHHPS--------PDHHQQHNNQ---HH-----HHHHP------------------
Tcas_Dsx        123 CSNP--------------------------------------------------------
Bmor_Dsx        142 GVSP--------------------------------------------------------
Ekue_Dsx        136 AVSP--------------------------------------------------------
Tdom_Dsx        149 SSSN------GV------------------------------------------------
Dmel_Dmrt99B    144 HGGIPSPPSDGFEASSTPNHHQQSQQQQQAHHQQHLSHPHQQSQRFNGNESDTDGRTRSE
Lcup_Dmrt99B    140 HAGIPSPPSDGLDSTNNSTHH----------------HTRMPFSNGNSGDSDSEMRIRAE
Tcas_Dmrt99B    140 TASDSSEP----------------------------------------------------
Bmor_Dmrt99B    112 -------------------HH----------------YADMP------------------
Bmut_Dmrt1      147 SSQPPPAS----------------------------------------------------
Drer_Dmrt1      109 PSPASSSA----------------------------------------------------
Mmus_Dmrt1      163 SAQPP-------------------------------------------------------
Xlae_Dmrt1      121 TQTTSTPT----------------------------------------------------

                421 .......430.......440.......450.......460.......470.......480
Haxy_Dsx        121 ---------------------------------------------------PK-------
Dmel_Dsx        306 -----------------TTV-------------------------------PT-------
Lcup_Dsx        270 -------------HPHSSAV-------------------------------PP-------
Tcas_Dsx        127 ---------------------------------------------------PP-------
Bmor_Dsx        146 -------------YAPPPSV-------------------------------PP-------
Ekue_Dsx        140 -------------YAPQPPLS-----------------AP-----------PP-------
Tdom_Dsx        155 ------------VVAVPSRL-------------------------------PP-------
Dmel_Dmrt99B    204 HLSVGFSPTRTELDESPVSKRGAALSNETDQDTGSESGSP--------SSPRPKVAGLFN
Lcup_Dmrt99B    184 RLSMGFSPDRTEV-ESPGSKR-ARLSNETDQDTGSES-AP--------SSPHPKGSNYHN
Tcas_Dmrt99B    148 --------------SSPTSKR-PRINVEDCSLEGSDS-EPEDLKKSRQSSPVP-------
Bmor_Dmrt99B    119 -------------DGSPVQKR-ARITEVC-----STSPSP--------INEQPRDP---K
Bmut_Dmrt1      155 -------------TPSTAAPGPGYSCFFF-------------------------------
Drer_Dmrt1      117 -------------TASPTNLGSRSMLSLS-------------------------------
Mmus_Dmrt1          ------------------------------------------------------------
Xlae_Dmrt1      129 -------------SGSTASSEGKVLIQEI-------------------------------
```

```
               481 .......490.......500.......510.......520.......530.......540
Haxy_Dsx       123 IMRTMSPL-AEPPTTSSSMGAVAQSTD---------------------------------
Dmel_Dsx       311 -PAQSLEGSCDSSSPS---------------PSSTSGAAILPISVSVNR---KNGANVPL
Lcup_Dsx       279 -TAQSVDSSCDSSSPS---------------PSSTSGAISLPVNRKIVPEHHQNGADMSI
Tcas_Dsx       129 AIRKMTPVPAVPSSTSVNIGTIAQSTD---------------------------------
Bmor_Dsx       155 -PPTMPPL-IPPPQPH---------------YWWPGAFPVSPGHVSEQRLS-QEGNIKAV
Ekue_Dsx       152 -PPNMPPL-LPPPQP-------------------------------------------AV
Tdom_Dsx       165 -TARPVKLGIPPSSGH----------------------PAMPPNVTVGE-----------
Dmel_Dmrt99B   256 LTASLSPARTGPPSSP--ESDLDVDSAPDEA---------TPENLSLKKEDSQSPPNTPA
Lcup_Dmrt99B   233 -NGNLSPARTGPPSSP--ESDLDVDSAPDEA---------TPENLSLKKEDSTSPPHTPS
Tcas_Dmrt99B   185 APAPSAPTPSPEPQTSP-DPDLDVEEDTQSE---------APENLSLKKPSSPETPPQPT
Bmor_Dmrt99B   149 TPAPTPPLATPPPRSPS-DHELNVEEELEES-----EPSLTPENLSMKESR-EEMPVKKE
Bmut_Dmrt1     171 -----------PAVTS--RGHVENTPDLVSD---------STYYSSFYQP-----SLFPY
Drer_Dmrt1     133 -----------PAMSS--RGHTDCTSDLMVD---------ASYY-NLYQPT----PYSSY
Mmus_Dmrt1         ------------------------------------------------------------
Xlae_Dmrt1     145 -----------PSITS--RGHMESTSDLVMD---------SPYYSNFYQP-----PLYPY

               541 .......550.......560.......570.......580.......590.......600
Haxy_Dsx       149 -----LLEDCQK-------LLERFKY----------------------------------
Dmel_Dsx       352 -GQDVFLDYCQK-------LLEKFRY----------------------------------
Lcup_Dsx       323 ---DLILDYCQK-------LIEKFGY----------------------------------
Tcas_Dsx       156 -----LLEDCQK-------LLERFKY----------------------------------
Bmor_Dsx       197 -PSETLVENCHR-------LLEKFHY----------------------------------
Ekue_Dsx       167 -SLENLVENCQK-------LLEKFHY----------------------------------
Tdom_Dsx       191 -NVEVLKDSLHA-------LLDMFRL----------------------------------
Dmel_Dmrt99B   305 -ENLHLLRSFSSSHAQGFLPYHHTQFLAAAGLPAHHHP--AAHSPHHQQQQQQQQQQQNH
Lcup_Dmrt99B   281 NDSMHMLRSFNNGNAQGFMPYHHTQFLAAAAASAHSHAQHAQHSPHHPQQPQMP------
Tcas_Dmrt99B   235 ---------------QNFIPYQQFAF----------------------------------
Bmor_Dmrt99B   202 ---------------EEKWENNEFKY---------------------------KMFRRKD
Bmut_Dmrt1     204 -------------------YNNLYNY----------------------------------
Drer_Dmrt1     166 -------------------YSNLYNY----------------------------------
Mmus_Dmrt1         ------------------------------------------------------------
Xlae_Dmrt1     178 -------------------YNNLYNY----------------------------------

               601 .......610.......620.......630.......640.......650.......660
Haxy_Dsx       163 -PWE------------------MMPL------------MHAILKGARADLEEASRRIDEG
Dmel_Dsx       370 -PWE------------------LMPL------------MYVILKDADANIEEASRRIEEA
Lcup_Dsx       339 -PWE------------------MMPL------------MYVILKDAGVDIDEASKRIEEG
Tcas_Dsx       170 -PWE------------------MMPL------------MYAILKDARADLEEASRRIDEG
Bmor_Dsx       215 -SWE------------------MMPL------------VLVIMNYARSDLDEASRKIYEG
Ekue_Dsx       185 -SWE------------------MMPL------------VLVILNYAGSDLDEASRKIDEA
Tdom_Dsx       209 -PLE------------------TLPL------------IYVVLKDARSDVKEASNRIMEA
Dmel_Dmrt99B   362 LPQHHQQQQQQQQQQQRSPIDVLMRV----FPNRRRSDVEQLMQRFRGDVLQAMECMLAG
Lcup_Dmrt99B   335 -PQH-----PSASQQQRSPVDVLMRV----FPNRRRSDVEQLLQRYRGDVLQTMEAMISG
Tcas_Dmrt99B   246 -PPF-----QPQYPAQRSPIDVLMRV----FPGKRRSDVEALLQRCKGDVVQAMEMMVSG
Bmor_Dmrt99B   220 LPEP----AHEESQYQKSPVDVLLKV----FPRRSRQEIEAILARCKGDVVAAMDSMVNG
Bmut_Dmrt1     211 -PQY-----PMALAADSSSGDVGNPLGGSPVKNSLRSLPAPYVPGQTGNQWQMKNS----
Drer_Dmrt1     173 -QQY-----QM--------------------------------PSGNG------------
Mmus_Dmrt1         ------------------------------------------------------------
Xlae_Dmrt1     185 -PPY-----QMAMAAESTSGNDMGGISGPPLKNNHRNHPAAYVPSQSGNQWQMKNR----

               661 .......670.......680.......690.......700.......710.......720
Haxy_Dsx       192 KEA--EI----LLEFCQRIRDK---FQISWRMVALVNVILKQANEDQEEAWRQIDEAFLE
Dmel_Dsx       399 RVEI--------------------------------------------------NRTVAQ
Lcup_Dsx       368 IQVLKQY----NLNIYDGNELRK---------------LKTERRYENHLARSECDETIKQ
Tcas_Dsx       199 RDT--EI----LLDFCQRLKDK---FQLSWKMISLVDVILKYA-KDQDEAWRQIDEAFLE
Bmor_Dsx       244 KMIVDEYARKHNLNVFDGLELRNSTRQ---------------------------------
Ekue_Dsx       214 HWMVHQW----RLSLCSLLQAR---KE---------------------------------
Tdom_Dsx       238 QAEL--------------------------------------------------------
Dmel_Dmrt99B   418 EDL---------------------------------------GQTPPQVP-----PSPP-
Lcup_Dmrt99B   385 EDIL-----------------------------------TNSTNSPPNVP-----PSPP-
Tcas_Dmrt99B   296 SHE---------------------------------------------------DATPP-
Bmor_Dmrt99B   272 PEP-----------------------------------------NPYHVT----QESPPY
Bmut_Dmrt1     261 -----------------------------------------ENR---HAV------SSQ-
Drer_Dmrt1     183 -------------------------------------------RLSSHNV------SPQ-
Mmus_Dmrt1         ------------------------------------------------------------
Xlae_Dmrt1     235 -----------------------------------------ENRFPGHSG------SSQ-
```

```
                 721 .......730.......740.......750.......760.......770.......780
Haxy_Dsx         243 VRTWAAVEAARSA-----------YRHIPYAGFYSTATALYHPHMYLPAIPTYHS-----
Dmel_Dsx         409 I------------------Y----YNYYTPMAL------VNGAPMYL-TYPSIEQGRY--
Lcup_Dsx         409 IR---LKEATEQLNQLTQTY----YNYQRYGTL---------PPAYW-AYPSIQLGRTIW
Tcas_Dsx         249 IRALAAVEAARYT-----------YHHIPYSGLYPNAATAIYPPVYLPSMSMYH------
Bmor_Dsx         271 ------------------------YGL---------------------------------
Ekue_Dsx         234 ------------------------YSMSC-----CSPRFVIAPE-YA-------------
Tdom_Dsx         242 -RSMALREAARVIHYPGPYY----YNYYP-------------PTPYLPTPPSIDL-----
Dmel_Dmrt99B     433 ---FPMKSAFSPLVPP-SVFGSPTHRYHPFMQA-HAKRFLTAP--YA-------------
Lcup_Dmrt99B     404 ---FPLKSAFSPLVPPAAVFGSPTHRYPPFMQA-HAKRFLTAP--YA-------------
Tcas_Dmrt99B     304 -------SAFSPLGPP-TNF----HRFSP------SRRFLSAP--YA-------------
Bmor_Dmrt99B     287 LQNYQSKSAFSPLSNQ-------SFKFSP------SRRFLTPP--YS-------------
Bmut_Dmrt1       270 ---YRM------------------HSYYP-------------PPSYL-------------
Drer_Dmrt1       193 ---YRT------------------HSYYS-------------S--YL-------------
Mmus_Dmrt1           ------------------------------------------------------------
Xlae_Dmrt1       247 ---FRM------------------HSYYP-------------P--YL-------------

                 781 .......790.......800.......810.......820.......830.......840
Haxy_Dsx         287 -----------------GADLLPTVPSRS-PPLPQAPSLTPPQSN--AVLRPGSRA----
Dmel_Dsx         438 ----GAHFTHLPLTQICPPTPEPLALSRS----PSSPSGPSAVHNQ-KPSRPGSSNG---
Lcup_Dsx         452 TELPTPHFAAIIPPHSAPTPEEPLTLSRA----STSPS---------KISRSGSSSICGE
Tcas_Dsx         292 -----------------PATLLGSVPTST------SPSHSPPIVP--RAIRPSSRA----
Bmor_Dsx             ------------------------------------------------------------
Ekue_Dsx         251 -----------------PLLPLPLTTQRP----SPPPAHL--------------------
Tdom_Dsx         279 -----------------PSYPPPLIPHVS-TGLAAAPE----------------------
Dmel_Dmrt99B     473 -----------------GTGYLPGVLSAA------------------DIEQSESNG----
Lcup_Dmrt99B     445 -----------------GTGYLPGVINPA------------------DVEQSESN-----
Tcas_Dmrt99B     331 -----------------GTGYLPTVIRPP-------PDYLSMVGSVHDIYSSDKT-----
Bmor_Dmrt99B     319 -----------------GTGYLPTVIRPP-------PEYLSSFMPPSELMYGRQL-----
Bmut_Dmrt1       283 -----------------GQSMSQ------IFTFEDSASYSEAKASVFSPPSSQDS-----
Drer_Dmrt1       204 -----------------SQGLGA-ACVQP-------STCPEPKAAAFS-DGAQDS-----
Mmus_Dmrt1           ------------------------------------------------------------
Xlae_Dmrt1       258 -----------------GQSVPNPACVPPFLTFEEIPSYSEAKASVLSPPSSQDS-----

                 841 .......850.......860.......870.......880.......890.......900
Haxy_Dsx             ------------------------------------------------------------
Dmel_Dsx         486 TVHSAASPTMVTTMATTSSTPTLSRRQRSRSATPTTPPPPPPAHSSSNGAYHHGHHLVSS
Lcup_Dsx         499 SITATSTPTPTTT--TTPSAGVIAAAAAAAAAAAAT------------------------
Tcas_Dsx             ------------------------------------------------------------
Bmor_Dsx             ------------------------------------------------------------
Ekue_Dsx             ------------------------------------------------------------
Tdom_Dsx         299 ------SPRLIDGRAHTNTSTSAS------------------------------------
Dmel_Dmrt99B     494 --AGGIGLDRTSNAGDSQD-----------------------------------------
Lcup_Dmrt99B     465 ---GGASVDRNSNAGDSQD-----------------------------------------
Tcas_Dmrt99B     362 ---SASSPGSNTSSDKTSYSE---------------------------------------
Bmor_Dmrt99B     350 ---QVPSPGTSPTSDNTNNDGFSD------------------------------------
Bmut_Dmrt1       315 ---GLVSLPSSSPIGNESTKAVLDCESASEPSNFAVAP-------------------IIE
Drer_Dmrt1       233 -----VSISSMINAENK-----LECESSSESGSFSVDS-------------------IIE
Mmus_Dmrt1           ------------------------------------------------------------
Xlae_Dmrt1       296 ---GVISLSSNSPVSNESTKAVAEQEPNSESSLFTVTT-------------------AAE

                 901 ....
Haxy_Dsx             ----
Dmel_Dsx         546 TAAT
Lcup_Dsx             ----
Tcas_Dsx             ----
Bmor_Dsx             ----
Ekue_Dsx             ----
Tdom_Dsx             ----
Dmel_Dmrt99B         ----
Lcup_Dmrt99B         ----
Tcas_Dmrt99B         ----
Bmor_Dmrt99B         ----
Bmut_Dmrt1       353 EDE-
Drer_Dmrt1       264 GATK
Mmus_Dmrt1           ----
Xlae_Dmrt1       334 NGE-
```
