# Supplementary figures and images for: Sex‐Specific doublesex Regulation Targeting the Color‐Patterning Gene h Underlies the Evolution of Wing Sexual Dimorphism in the Harlequin Ladybug Harmonia axyridis
Source: Evol Dev. 2026 Jan 6;28(1):e70028. doi: 10.1111/ede.70028 (PMC12771471; doi:10.1111/ede.70028)

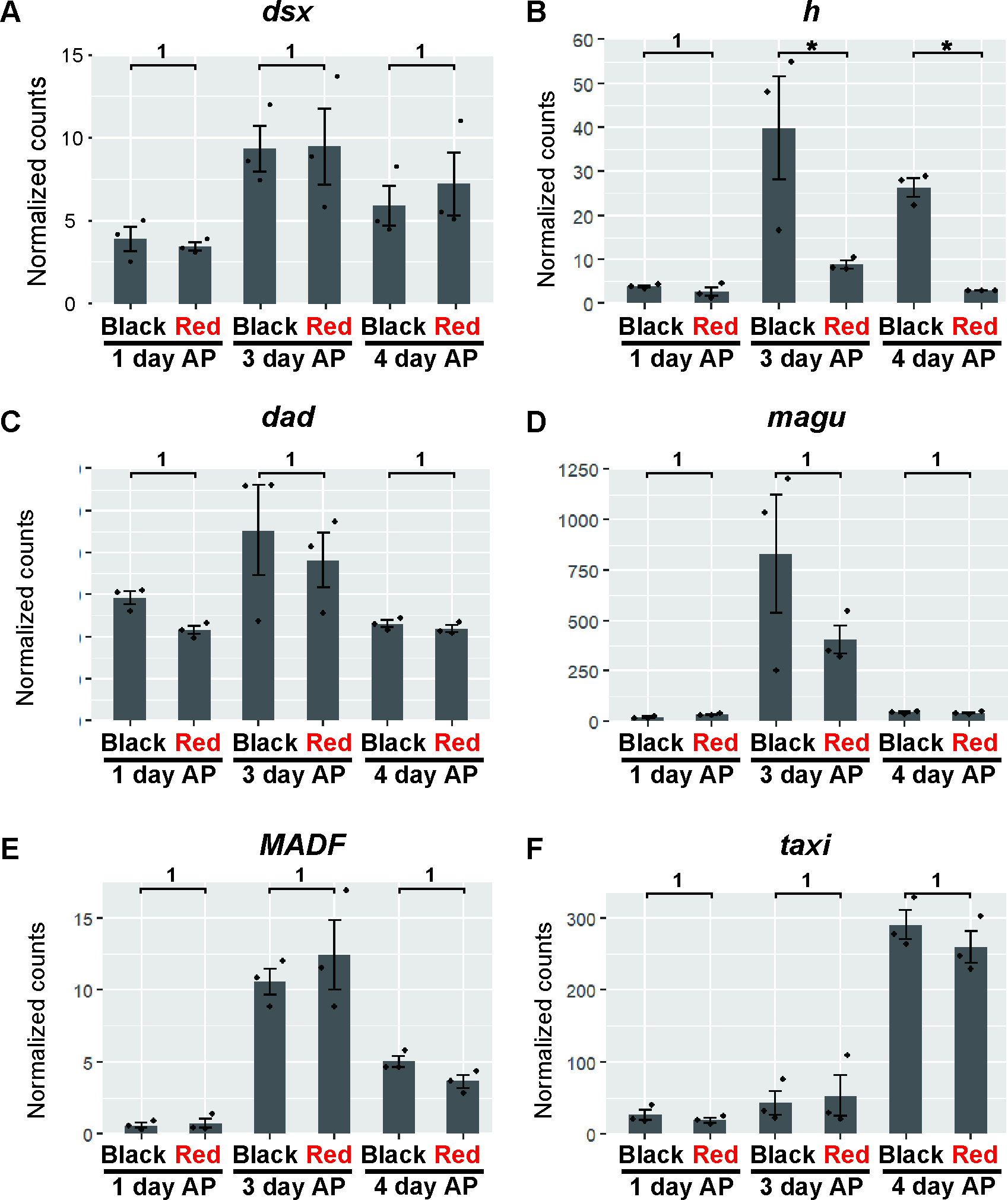

Supplement: Supplementary file 3 — Fig. S3. Developmental expression profile of dsx and downstream regulatory factors in the pupal elytra. Expression of (A) dsx, (B) h, (C) dad, (D) magu, (E) MADF, and (F) taxi in the red (Red) and black (Black) regions of the pupal wing at different developmental stages (1, 3, and 4 days after pupation [days AP]). dsx expression was detected as early as 1 day AP and was observed in both red and black regions of the elytra throughout development. h showed higher expression in the black region 3 and 4 days after pupation (B), whereas the other downstream regulators showed comparative expression between red and black regions at each stage (C–F). FDR‐adjusted P values (q values) from the Wald test are shown above the bars (*: q < 0.05). [file EDE-28-e70028-s003.tif]

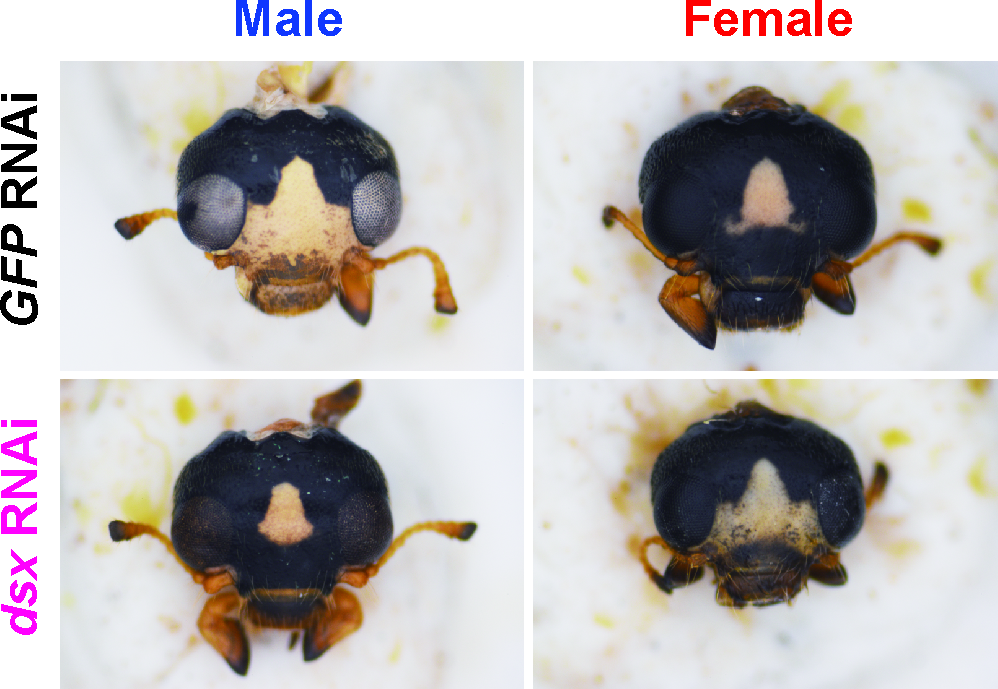

Supplement: Supplementary file 4 — Fig. S4. Effects of dsx knockdown on head pigmentation. Comparison of the head color in RNAi‐treated individuals between dsx RNAi and GFP RNAi treatments in both sexes. In wild‐type individuals, the frons (the frontal surface of the head) is typically white in males and black in females. We found that dsx RNAi‐treated males exhibited ectopic frons pigmentation, which was absent in wild‐type males, whereas dsx RNAi‐treated females showed reduced pigmentation compared to wild‐type females. [file EDE-28-e70028-s007.tif]

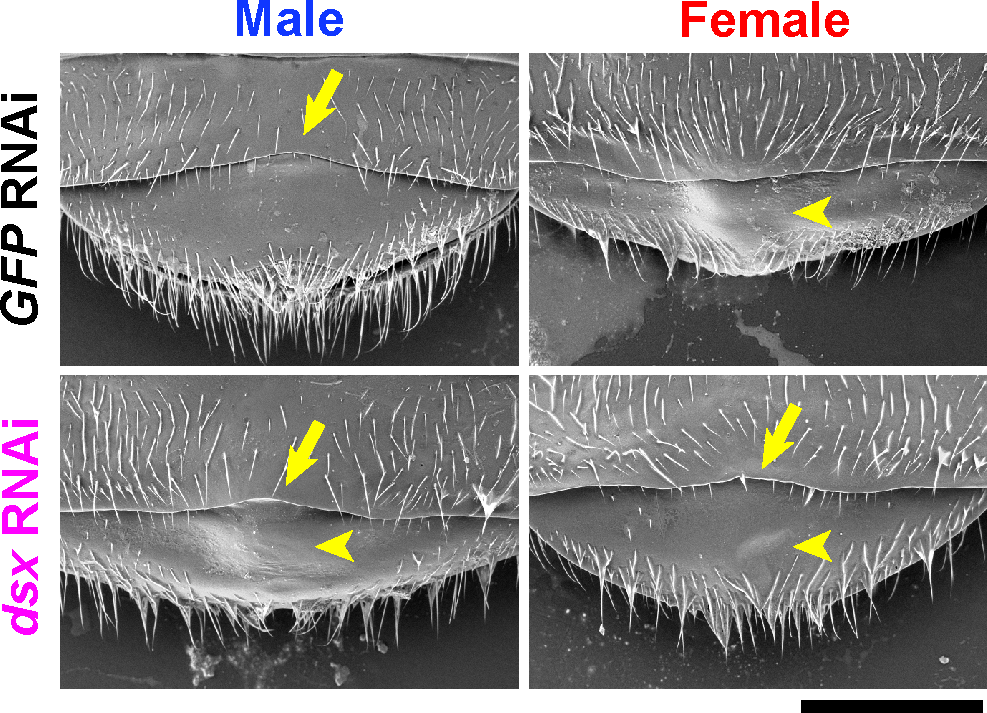

Supplement: Supplementary file 5 — Fig. S5. Effects of dsx knockdown on hypopygial morphology. Comparison of the hypopygial morphology in RNAi‐treated individuals between dsx RNAi and GFP RNAi treatments in both sexes. In wild‐type individuals, the posterior margin of the fifth abdominal segment is concave in males (arrow), whereas it is flat in females, with a median protrusion on the sixth segment present only in females (arrowhead). We observed morphological abnormalities in both male and female dsx RNAi individuals. Specifically, both dsx RNAi males and females showed reduced concavity of the fifth segment (arrow), and a small protrusion on the sixth segment (arrowhead). [file EDE-28-e70028-s006.tif]

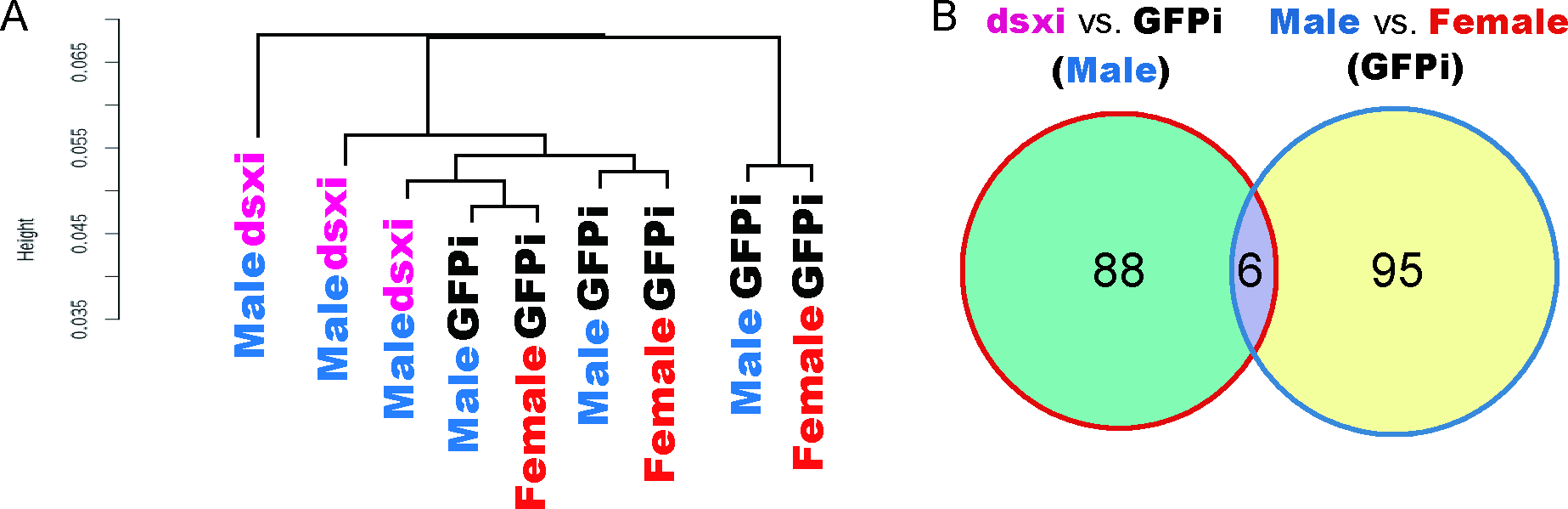

Supplement: Supplementary file 6 — Fig. S6. Comparison of transcriptomes between dsx RNAi‐treated males and controls (males/females). (A) Hierarchical clustering analysis of the transcriptome data showing overall similarity or divergence in gene expression among the three groups. (B) Venn diagram showing the number of differentially expressed genes (DEGs) between dsx RNAi‐treated males (dsxi) and control RNAi‐treated males (GFPi), and between control RNAi‐treated males and females (control RNAi, GFPi). [file EDE-28-e70028-s001.tif]

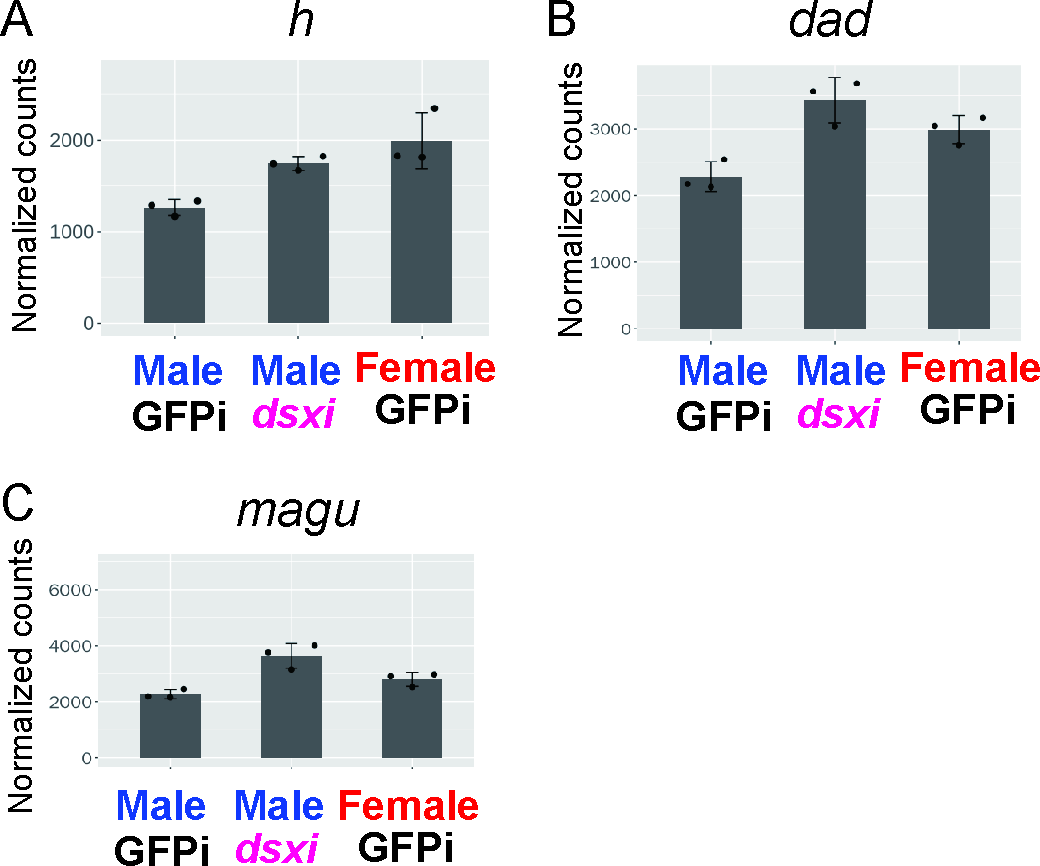

Supplement: Supplementary file 7 — Fig. S7. Upregulation of TGF‐β‐associated genes in dsx RNAi in males. Normalized expression levels of three genes—(A) h, (B) dad, and (C) magu—were extracted from transcriptome data. Expression profiles are shown for dsx RNAi‐treated males (dsxi), control RNAi‐treated males (Male GFPi), and control RNAi‐treated females (Female GFPi). [file EDE-28-e70028-s008.tif]

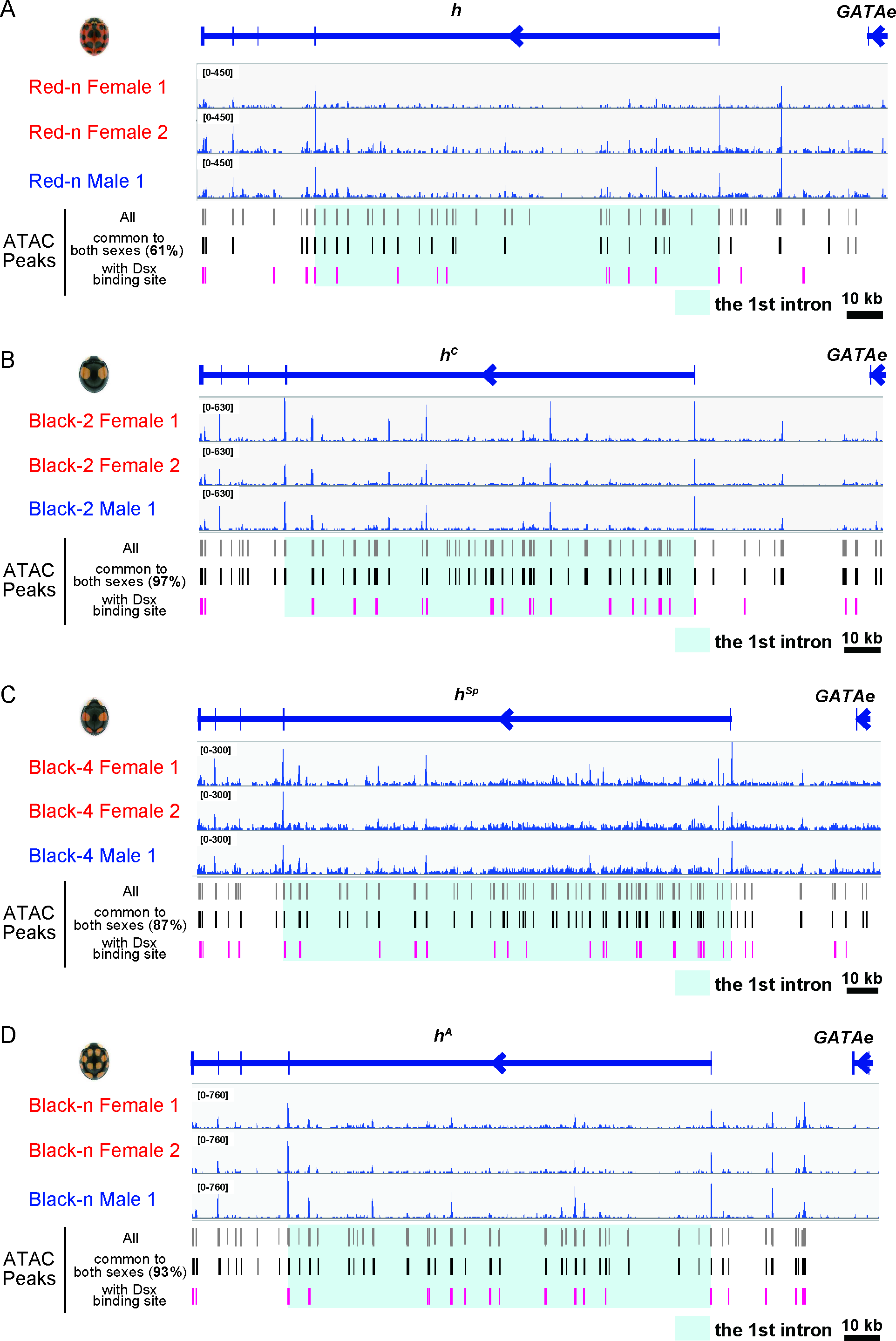

Supplement: Supplementary file 8 — Fig. S8. Distribution of Dsx binding motifs and ATAC‐seq peaks at the h locus in females and males of the four major color pattern alleles. (A–D) Distribution of chromatin accessibility and Dsx binding motifs at the h locus for four representative h alleles: (A) Red‐nSpots, (B) Black‐2Spots, (C) Black‐4Spots, and (D) Black‐nSpots. Each panel consists of: (Line 1) Schematic of the genomic structure. (Lines 2‐4) ATAC‐seq read coverage: (2) female replicate 1, (3) female replicate 2, (4) male. (Lines 5–7) Distribution of ATAC‐seq peaks: (5) merged peaks from females and males (“All”), (6) peaks shared by both sexes (“common to both sexes”), and (7) peaks that contain Dsx binding motifs (“with Dsx motif”). [file EDE-28-e70028-s005.tif]
